# Reported frequency of physical activity in a large epidemiological study: relationship to specific activities and repeatability over time

**DOI:** 10.1186/1471-2288-11-97

**Published:** 2011-06-22

**Authors:** Miranda EG Armstrong, Benjamin J Cairns, Jane Green, Gillian K Reeves, Valerie Beral

**Affiliations:** 1 Cancer Epidemiology Unit, University of Oxford, Richard Doll Building, Old Road Campus, Oxford OX3 7LF, UK

## Abstract

**Background:**

How overall physical activity relates to specific activities and how reported activity changes over time may influence interpretation of observed associations between physical activity and health. We examine the relationships between various physical activities self-reported at different times in a large cohort study of middle-aged UK women.

**Methods:**

At recruitment, Million Women Study participants completed a baseline questionnaire including questions on frequency of strenuous and of any physical activity. About 3 years later 589,896 women also completed a follow-up questionnaire reporting the hours they spent on a range of specific activities. Time spent on each activity was used to estimate the associated excess metabolic equivalent hours (MET-hours) and this value was compared across categories of physical activity reported at recruitment. Additionally, 18,655 women completed the baseline questionnaire twice, at intervals of up to 4 years; repeatability over time was assessed using the weighted kappa coefficient (κ_weighted_) and absolute percentage agreement.

**Results:**

The average number of hours per week women reported doing specific activities was 14.0 for housework, 4.5 for walking, 3.0 for gardening, 0.2 for cycling, and 1.4 for all strenuous activity. Time spent and the estimated excess MET-hours associated with each activity increased with increasing frequency of any or strenuous physical activity reported at baseline (tests for trend, P < 0.003), although the associations for housework were by far the weakest (Spearman correlations, 0.01 and -0.03 respectively for housework, and 0.11-0.37 for all other activities). Repeatability of responses to physical activity questions on the baseline questionnaire declined significantly over time. For strenuous activity, absolute agreement was 64% (κ_weighted _= 0.71) for questionnaires administered less than 6 months apart, and 52% (κ_weighted _= 0.51) for questionnaires more than 2 years apart. Corresponding values for any physical activity were 57% (κ_weighted _= 0.67) and 47% (κ_weighted _= 0.58).

**Conclusions:**

In this cohort, responses to simple questions on the frequency of any physical activity and of strenuous activity asked at baseline were associated with hours spent on specific activities and the associated estimated excess MET-hours expended, reported 3 years later. The weakest associations were with housework. Agreement for identical questions asked on two occasions about the frequency of physical activity decreased over time.

## Background

Reliable assessment of physical activity over a long period is important for examining the effects of physical activity on health in cohort studies. For large-scale prospective studies, use of objective measures of physical activity, such as accelerometers and calorimetry, are impractical and questionnaires have become the method of choice for the assessment of physical activity [1-4]. Questionnaires are less likely than objective methods to interfere with usual physical activity patterns, allow the assessment of multiple variables with the same instrument, are relatively inexpensive, and are simple to administer and to score [3]. Yet, responses to questions are subjective, and questionnaires often ask about only a portion of overall physical activity, and are therefore subject to measurement error [1,3-7]. Self-report of socially sensitive information such as physical activity may also be subject to social desirability response bias, the tendency of individuals to respond to questions in a way that portrays them in a positive light [8,9].

In prospective studies, physical activity assessed at baseline may be used to investigate the influence of physical activity on health outcomes which occur during follow-up. However, baseline assessments do not necessarily represent physical activity during extended periods of follow-up, both due to measurement errors at the time of assessment, and because physical activity behaviours may change over time [10-13]. As with any exposure data that is subject to error [14], the result of differences between baseline physical activity data and typical physical activity behaviours during follow-up will generally be to underestimate associations between baseline physical activity and health outcomes.

The Million Women Study is a prospective cohort study of 1.3 million middle-aged women recruited in the UK. It was designed to assess the effects of lifestyle factors on a variety of disease outcomes in women [15]. Since follow-up is planned to span many years, it is important to establish how measurement errors and changes since recruitment might affect associations with subsequent disease risk.

We evaluated the associations between overall frequency of physical activity reported at baseline, and various types of physical activity (walking, gardening, cycling, housework, and strenuous activities) reported 3 years after recruitment. These findings can be used to assess how well women's responses to baseline questions on physical activity are associated with their reported levels of physical activity over an extended period of follow-up. To investigate the combined effects of reporting error and changes in physical activity behaviours over time, we also examined the repeatability of responses to the same questions on frequency of physical activity by women who completed the baseline questionnaire twice with intervals between them ranging from less than 6 months to more than 2 years. These measures can be used to assess the potential attenuation of disease associations with physical activity as measured at baseline, for example through the use of methods similar to those used to correct for "regression dilution bias" [14,16].

## Methods

### Data collection

The Million Women Study is a prospective cohort study of middle aged women in the UK. Details of the design and methods of the Million Women Study have been published elsewhere [15]. From 1996-2001, women were recruited via national breast screening programmes in England and Scotland. In total, 1.3 million respondents, aged 56 years on average, completed a baseline questionnaire which included questions on physical activity, lifestyle, medical history, and socio-demographic factors. Permission to conduct the study was granted by the Anglia and Oxford Multi-Centre Research Ethics Committee.

On the baseline questionnaire all women were asked "How often do you do any strenuous exercise? (that is, enough to cause sweating or a fast heart beat)", and after the first 9% had completed the questionnaire an additional question was added: "How often do you do any exercise?". To each of these questions, study participants were given 6 options to respond: 'Rarely/never', 'less than once a week', 'once a week', '2-3 times a week', '4-6 times a week', 'every day'. No distinction was made between frequencies of physical activity in summer and winter. Of the 1.3 million participants, 18,655 (1.4%) completed the recruitment questionnaire twice (17,617 women provided repeated data for strenuous activity, and 12,748 provided repeated data for any activity).

A follow-up questionnaire was sent to study participants approximately three years after recruitment with an overall response rate of 65%. Over 600,000 women responded to questions asking: "About how many hours each week do you spend doing: housework, gardening, walking, cycling, any work or exercise causing sweating or a fast heartbeat". These questions were based on session-based physical activity measures used in the International Physical Activity Questionnaire [17] and the Active Australia Survey [18]. Respondents were asked to report separately on activity durations during summer and winter, except for housework. A total of 589,896 women responded to physical activity questions on both baseline and follow-up questionnaires, and were included in the analyses comparing the two sets of responses.

### Frequency of physical activity at baseline and reported specific activities at follow-up

The mean estimated excess energy expenditure for strenuous activity and various specific activities reported at follow-up (derived from hours spent performing these activities), were compared across categories of frequency of strenuous and any physical activity at baseline. Spearman correlation coefficients and P-values for trend were also calculated for these comparisons.

To estimate excess energy expenditure, metabolic equivalents (METs) were assigned to each activity according to the compendium of physical activities published by Ainsworth et al., [19]. By definition, a MET is the ratio of the metabolic rate required by a given work task, to the standard resting metabolic rate obtained while sitting quietly [19]. Housework was estimated at 3.0 METs, gardening at 4.0 METs, and walking at 3.5 METs. Cycling and any work or exercise causing sweating or a fast heartbeat, were estimated at 8 METs. Multiplying by these values provides an estimate of the gross metabolic cost of each activity, i.e. the sum of the resting metabolic rate and the metabolic cost of the physical activity. In the present circumstance, however, the estimated net energy expenditure (the estimated energy expenditure associated with the given activity only) may be more appropriate, as in low intensity activities the resting metabolic rate accounts for a higher proportion of total estimated energy expenditure than in high intensity activities [20]. For example the proportion of energy expenditure attributed to resting metabolic rate for cycling is 12.5%, compared with 33% for the equivalent time spent doing housework. Because we calculated an estimate of excess energy expenditure (net cost of physical activity) attributed to physical activity only, beyond energy expenditure attributed to resting metabolic rate, one MET was subtracted from each multiplier prior to calculations. The MET values were multiplied by the number of hours reported for each type of activity to obtain energy expenditure as excess MET-hours, which include only additional activity above basal MET values. Reported summer and winter values were averaged to obtain the total time spent doing a reported activity. The calculation of excess MET-hours for strenuous activity at follow-up included hours of strenuous activity reported when answering the question asking about hours spent doing exercise causing sweating or a fast heartbeat. To avoid possible double counting and due to the similarity between the question asked at baseline ["How often do you do any strenuous exercise? (that is, enough to cause sweating or a fast heart beat)"] and that asked at follow-up ("About how many hours each week do you spend doing any work or exercise causing sweating or a fast heartbeat"), cycling was not added to the values reported for strenuous exercise at follow-up. Hours and excess MET-hours for all of the various activities reported at follow-up (housework, gardening, walking, cycling, and any strenuous exercise) were summed together to provide an aggregate physical activity value. However, as only pre-specified activities were reported at follow-up, this value should not be assumed to represent total activity.

### Repeatability over time of reported frequency of physical activity

Agreement was assessed between women's first and repeat responses to the baseline questions on frequency of any and of strenuous physical activity. The percentage absolute agreement reflects all changes in reported activity levels between first and repeat responses. As the categories of reported frequency of physical activity per week were ordinal (ordered from least frequent to most frequent activity), the kappa coefficient with quadratic weighting (κ_weighted_) was also assessed [21,22]. The kappa coefficient with quadratic weighting is equivalent to an intraclass correlation coefficient [22] and allows for the fact that a change from category 1 to category 2 (for example) reflects closer agreement over time than a change from category 1 to category 6. Kappa values above 0.80 are taken to indicate excellent agreement, between 0.61 - 0.80 substantial agreement, between 0.41 - 0.60 moderate agreement, between 0.21 - 0.40 fair agreement, and lower than 0.20 poor agreement [23].

The percentage of women reporting each frequency of strenuous or of any physical activity and the percentages of women reporting higher, lower or the same amount of strenuous or of any physical activity between first and repeat baseline questionnaires were also calculated for the 4 time periods.

The data were also assessed for differences in reporting between first and repeat response according to seasonality. The kappa coefficient with quadratic weighting (κ_weighted_) was used to assess agreement over time for different seasonal scenarios including winter at first response compared to winter at repeat response, winter at first response compared to summer at repeat response, summer at first response compared to summer at repeat response, and summer at first response compared to winter at repeat response. For the purposes of this analysis, data reported during December through February were defined as winter, and data reported during June through August were defined as summer.

The STATA statistical package (version 10, Statacorp, Texas) was used for all analyses.

## Results

### Frequency of physical activity at baseline and reported specific activities at follow-up

Women reported spending a total of 1.4 (SD = 3.4) hours per week on average doing strenuous activity at follow-up. The number of hours per week reported doing strenuous activity at follow-up increased with the frequency of strenuous activity reported at baseline 3 years earlier (Table 1). Women reporting no strenuous activity at baseline reported 0.8 hours per week on average of strenuous activity at follow-up, while those reporting daily strenuous activity at baseline reported 3.5 hours per week on average at follow-up. Total excess MET-hours for strenuous activity, calculated from the number of hours spent participating in strenuous activity at follow-up, was associated with baseline frequency of strenuous activity (Spearman correlation 0.37, P < 0.0001 for trend; Table 1). Similar associations were seen when excess MET-hours per week for an aggregate of various activities (walking, cycling, gardening, housework, and exercise causing sweating or a fast heartbeat) reported at follow-up were compared with the frequency of any physical activity reported at baseline (Spearman correlation 0.22, P < 0.0001 for trend; Table 1). On average, women reported spending 23.1 (SD = 14.6) hours per week at follow-up walking, cycling, doing gardening, doing housework, or doing exercise causing sweating or a fast heartbeat. Women who reported no activity at baseline reported an average of 21.0 hours per week of various activities at follow-up, whereas women reporting being active daily at baseline reported an average of 27.0 hours per week of various activities at follow-up.

**Table 1:** Excess MET-hours ^a ^for activity at follow-up in relation to baseline frequency of activity

					
		**Hours and excess MET-hours ^a ^per week spent doing various ** **activities reported ~3 years after baseline**			
	
		**Strenuous activity ^b^**		**Aggregate of various activities ^c^**	
**Frequency of strenuous physical** ** activity reported at baseline**	**Number ** **of women**	**hours ** **mean**	**MET-hours ^a ^** **mean (SD)**	**hours** ** mean**	**MET-hours ^a^** **mean (SD)**
	
Never	261,857	0.8	5.6 (21.3)	21.7	52.4 (39.0)
< Once per week	79,962	1.1	7.8 (20.2)	22.7	57.3 (38.0)
Once per week	115,573	1.5	10.7 (20.5)	23.4	61.5 (38.5)
2 to 3 times per week	96,415	2.4	16.5 (25.0)	24.8	69.5 (42.5)
4 to 6 times per week	19,394	3.4	23.9 (34.1)	27.2	81.3 (50.6)
Daily	16,431	3.5	24.3 (50.8)	31.1	90.6 (69.1)
Correlation coefficient			0.37	0.12	0.22
P for trend			< 0.0001	< 0.0001	< 0.0001
					
**Frequency of any physical** ** activity reported at baseline**					
					
Never	107,346	0.8	5.8 (24.5)	21.0	50.3 (41.4)
< Once per week	49,073	0.9	6.6 (21.2)	20.6	50.9 (37.8)
Once per week	92,317	1.2	8.1 (19.5)	21.3	53.9 (37.0)
2 to 3 times per week	149,138	1.6	11.4 (20.6)	22.7	60.0 (37.5)
4 to 6 times per week	57,585	2.0	13.8 (24.0)	24.2	66.0 (39.6)
Daily	134,437	1.7	12.0 (30.0)	27.0	71.2 (49.0)
Correlation coefficient			0.17	0.17	0.22
P for trend			< 0.0001	< 0.0001	< 0.0001

^a ^Excess MET-hours: metabolic equivalent hours of energy expenditure in excess of basal metabolic rate; see Methods for details.

^b ^Reported hours and estimated excess MET-hours ^a ^spent doing exercise causing sweating or a fast heartbeat

^c ^Reported hours and estimated excess MET-hours ^a ^spent doing walking, cycling, gardening, housework, and exercise causing sweating or a fast heartbeat

On average women reported 14 hours of housework, 4.5 hours of walking, 3.0 hours of gardening, and 0.2 hours of cycling per week. The number of hours per week reported doing walking, cycling and gardening at follow-up increased with the frequency of strenuous and of any activity reported at baseline 3 years earlier (Table 2). The corresponding estimated excess MET-hours are also given in Table 2. Walking, gardening and cycling showed stronger associations with reported frequency of any and strenuous activity (Spearman correlations 0.11-0.31, and all P < 0.0001 for trends) than did housework, which despite statistically significant trends was very poorly correlated with frequency of both strenuous and any activity (Spearman correlations -0.03 and 0.01, and P < 0.0001 and P = 0.002 for trends, respectively).

**Table 2:** Various reported physical activities at follow-up in relation to frequency of activity at baseline

	Hours and excess MET-hours ^a ^per week spent doing various activities reported ~3 years after baseline							
	**walking**		**cycling**		**gardening**		**housework**	
	**hours** ** mean**	**MET-hours** ** mean (SD)**	**hours** ** mean**	**MET-hours** ** mean (SD)**	**hours** ** mean**	**MET-hours** ** mean (SD)**	**hours** ** mean**	**MET-hours ** **mean (SD)**
				
**Baseline reported frequency ** **of strenuous physical activity**		n = 589,632		n = 589,632		n = 589,632		n = 589,632
								
Never	3.9	9.7 (12.8)	0.1	0.9 (5.7)	2.4	7.3 (9.9)	14.4	28.8 (23.0)
< Once per week	4.4	11.1 (12.1)	0.2	1.5 (6.8)	3.1	9.4 (10.8)	13.8	27.5 (21.2)
Once per week	4.8	12.0 (12.4)	0.3	1.9 (7.7)	3.3	9.8 (11.0)	13.5	27.0 (21.1)
2 to 3 times per week	5.2	13.1 (13.2)	0.4	2.8 (9.8)	3.5	10.5 (12.1)	13.3	26.6 (21.0)
4 to 6 times per week	5.9	14.8 (14.9)	0.6	4.4 (13.1)	3.8	11.4 (13.9)	13.4	26.7 (21.6)
Daily	7.5	18.6 (20.4)	0.6	4.4 (16.5)	4.1	12.3 (15.7)	15.4	30.9 (25.7)
Correlation coefficient		0.18		0.15		0.15		-0.03
P for trend		< 0.0001		< 0.0001		< 0.0001		< 0.0001
								
**Baseline reported frequency** ** of any physical activity**		n = 589,896		n = 589,896		n = 589,896		n = 589,896
								
Never	3.2	7.9 (12.7)	0.1	0.6 (4.9)	2.2	6.7 (9.8)	14.7	29.3 (23.7)
< Once per week	3.3	8.2 (11.2)	0.1	0.9 (4.7)	2.6	7.9 (9.8)	13.7	27.3 (21.8)
Once per week	3.7	9.2 (11.3)	0.2	1.3 (6.1)	2.8	8.4 (10.0)	13.5	27.0 (21.6)
2 to 3 times per week	4.4	11.0 (11.5)	0.3	1.8 (7.6)	3.0	9.1 (10.6)	13.4	26.8 (20.8)
4 to 6 times per week	5.2	13.0 (12.1)	0.4	2.7 (9.1)	3.2	9.6 (11.2)	13.5	27.0 (20.5)
Daily	6.5	16.3 (15.9)	0.4	2.6 (10.9)	3.6	10.8 (13.0)	14.8	29.6 (23.3)
Correlation coefficient		0.31		0.11		0.14		0.01
P for trend		< 0.0001		< 0.0001		< 0.0001		0.002

^a ^Excess MET-hours: metabolic equivalent hours of energy expenditure in excess of basal metabolic rate; see Methods for details.

### Repeatability over time of reported frequency of physical activity

**Figure 1:**
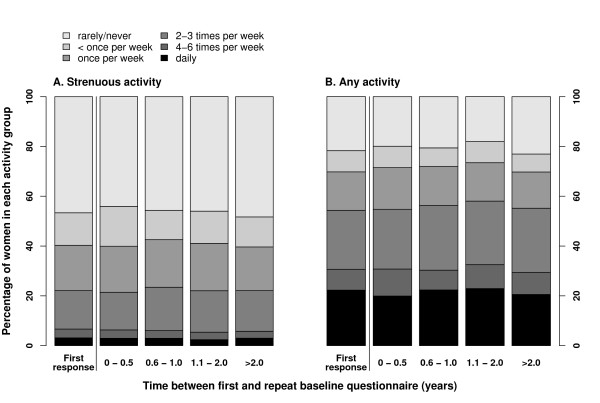
**Distribution of reported frequency of physical activity by time between first and repeat baseline questionnaires**. Shaded regions indicate proportions of women reporting the various frequencies of physical activity, either at baseline, or at repeat administration of the questionnaire stratified by the time between first and repeat responses.

**Figure 2:**
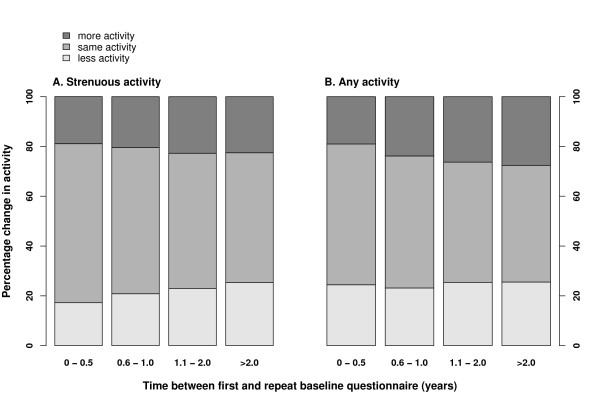
**Distributions of changes in reported physical activity by time between first and repeat baseline questionnaires**. Shaded regions indicate proportions of women reporting a frequency of physical activity on their repeat baseline questionnaire that was the same as, more than, or less than they reported at first administration. Women are stratified by the time between their first and repeat responses to the baseline questionnaire.

The overall distributions of reported frequency of strenuous or of any physical activity did not differ greatly according to the interval between first and repeat baseline questionnaires (Figure 1). However, agreement between first and repeat assessments of the frequency of strenuous and of any physical activity decreased over time (Figure 2 and Table 3). Agreement was better among women who completed the same questions less than 6 months apart (κ_weighted _= 0.67 and κ_weighted _= 0.71 for any activity and strenuous activity respectively) than among those who were asked more than 2 years apart (κ_weighted _= 0.58 and κ_weighted _= 0.51, respectively). Longer intervals between first and repeat assessments were associated with poorer agreement between responses (for strenuous and any activity, respectively, P = 0.03 and P = 0.05 for trends in weighted kappa statistics according to the interval between assessments). Analyses using further sub-categories of the interval between first and repeat baseline testing beyond 2 years did not alter these conclusions.

**Table 3:** Agreement of reported physical activity, by time between completing identical questionnaires

	**Number of** ** women**	**Agreement**	**Kappa (95% CI) with** ** quadratic weighting**
	
**Time between completing identical questions on** ** the frequency of strenuous activity** **(average time between the two)**			
			
≤ 6 months (0.3 years)	238	64%	0.71 (0.59 - 0.83)
> 6 months - 1 year (0.9 years)	1,224	59%	0.61 (0.55 - 0.67)
> 1 - 2 years (1.5 years)	7,002	54%	0.55 (0.53 - 0.57)
> 2 years (2.6 years)	9,153	52%	0.51 (0.49 - 0.53)
P for trend			0.03
			
**Time between completing identical questions on** ** the frequency of any activity** **(average time between the two)**			
			
≤ 6 months (0.2 years)	221	57%	0.67 (0.53 - 0.81)
> 6 months - 1 year (0.9 years)	1,171	53%	0.67 (0.61 - 0.73)
> 1 - 2 years (1.5 years)	6,044	48%	0.60 (0.58 - 0.62)
> 2 years (2.6 years)	5,312	47%	0.58 (0.56 - 0.60)
P for trend			0.05

P-values for trend in kappa were estimated by weighted least squares regression using the inverse of the variances of the kappa coefficients

The repeatability of questions on the frequency of strenuous or of any physical activity according to differences in the season of reporting was similar. Weighted kappa scores for baseline physical activity questions were: for first response in winter versus repeat response in winter, κ_weighted _= 0.52 (strenuous activity) and κ_weighted _= 0.57 (any activity); for first response in winter versus repeat response in summer κ_weighted _= 0.54 (strenuous activity) and κ_weighted _= 0.58 (any activity); for first response in summer versus repeat response in summer, κ_weighted _= 0.57 (strenuous activity) and κ_weighted _= 0.63 (any activity); for first response in summer versus repeat response in winter, κ_weighted _= 0.51 (strenuous activity) and κ_weighted _= 0.58 (any activity).

## Discussion

Our results from a cohort of middle-aged UK women showed that hours and corresponding estimated excess MET-hours for walking, cycling and gardening at follow-up were associated with the frequency of both any and strenuous physical activity reported at baseline about 3 years earlier. Mean estimated excess MET-hours for strenuous activity reported at follow-up also increased progressively with each increase in the frequency of strenuous physical activity reported at baseline. Similar relationships were found between any activity reported at baseline and an aggregate of various activities (walking, cycling, gardening, housework, and exercise causing sweating or a fast heartbeat) reported at follow-up. Hence, simple questions on the frequency of strenuous and any activity at baseline predict the estimated excess energy expenditure for strenuous and other specific activities, reported about 3 years later.

It has been suggested that older populations tend to be more sedentary, resulting in decreased between-person variability when compared to younger populations, and that this may reduce the power of a physical activity measurement instrument to discriminate between different activity levels [24]. It has also been proposed that measurement error associated with reported physical activity may increase as the proportion of total activity consisting of light intensity activities increases, as often occurs in older populations [25]. Social desirability bias may also influence the ability of some questionnaires to accurately assess self-reported levels of physical activity among middle-aged women [9].

Due to constraints on the length of the baseline questionnaire, simple, frequency-based questions were used to assess physical activity behaviours. At follow-up women reported on hours spent walking, cycling, doing gardening, doing housework, or doing exercise causing sweating or a fast heartbeat. Wareham et al. [26] showed that a simple four-level physical activity index, derived from questions asking the number of hours per week spent doing physical exercise, cycling and type of occupational physical activity, had high repeatability and was positively associated with objective measures of physical activity, and was therefore useful when ranking the physical activity levels of participants in large scale studies. In the Million Women Study, most women were aged 50 to 64 years at baseline. Only one fifth of women reported being in full-time work on the follow-up questionnaire, hence we did not include work effort.

Women reporting never being active at baseline reported taking on average 21.0 hours per week of various activities (walking, cycling, gardening, housework, and exercise causing sweating or a fast heartbeat) at follow-up. The lack of correlation between frequency of baseline activity and housework reported at follow-up may result from women not considering housework when asked to report on physical activity at baseline. However, domestic activities may account for a large proportion of total activity among these women. For example, for women in Britain aged 60 to 79 years, when domestic activities were not taken into account only 21% were classified as regularly active; whereas when domestic activities were included more than two thirds of these women met the requirements for achieving the recommended levels of physical activity [27]. It is also possible that some women who reported never being active at baseline were subject to a degree of physical impairment that prevented them from engaging in higher-intensity activities, but not necessarily low-intensity activities such as housework. No prospective information was obtained at baseline, however, which indicated levels of physical or functional impairment, and we were therefore unable to examine this hypothesis in more detail.

We also examined repeatability of women's responses to questions on frequency of physical activity over time. The overall distribution of responses to these questions was similar between first and repeat administrations of the same baseline questionnaire, regardless of the interval between these assessments. However, agreement between individual women's responses declined as this interval increased. This was more marked for strenuous activity. In terms of repeatability, our results are consistent with those previously reported [5,24,25,28,29], which have shown a decrease in the agreement of physical activity measures with greater time periods between initial and repeat testing. At the population level, a similar distribution across activity groups despite changes among individuals between first and repeat testing has also been reported [19-22].

Measurement errors in the assessment of long term activity patterns result from a combination of variability in answers when completing a questionnaire (resulting from difficulty recalling past activity, differing interpretations of the questions, social desirability response bias, and random reporting errors) and real changes in physical activity patterns over time [30]. Overall, these errors are likely to result in attenuation of estimates of associations between physical activity assessed at baseline and disease risk. This attenuation may be substantial and is likely to depend on the assessment instrument as well as participant characteristics [31]. Intensive assessments of physical activity may minimise some sources of error, but can be impractical in larger-scale studies over a long period [1,3,5].

Test/retest reliability of physical activity questionnaires is often examined over short time periods of weeks to a few months [32], but our findings underscore the importance of changes in physical activity over longer periods. The reduction in the repeatability of physical activity responses over time which we have observed is likely to reflect, at least in part, real changes in physical activity patterns. This type of measurement error is of great importance in prospective epidemiological studies, where risks of various health outcomes during an extended period of follow-up may be estimated according to baseline self-reported physical activity. Repeatedly measuring different aspects of physical activity over time permits the assessment of the potential magnitude of the attenuation of disease associations. This can be done, for example, by using methods analogous to those used when correcting for "regression dilution bias" [14,16].

Published surveys have reported evidence of changing physical activity levels in diverse populations in developed countries. An analysis of secular trends of physical activity levels in the adult population of the UK from 1991 to 2004 using data from the Health Survey for England, has shown a decline in occupational physical activity, but a progressive increase in sports participation in women aged 50 years and over [33]. Overall, in adults aged 50 to 64 years there was a significant increase from 42.9% to 47.1% (p < 0.001) for those meeting the current physical activity recommendations. Guthrie [11] showed that within a cohort of Australian-born women, aged 45 to 55 years at baseline in the Melbourne Women's Midlife Health Project, 14% increased their frequency of physical activity by two or more sessions per week and 12% decreased their activity by the same amount over a 3-year follow-up period. Similarly, among 20 to 59 year old adults from the Netherlands [12], 45% changed their level of physical activity over a ten year period. Similar patterns of change have also been observed in a range of age-groups studied in the UK [10], and the USA [13]. Our study had a longer mean interval between first and repeat baseline questionnaire administrations than many other studies, and is likely to be more conservative in assessing agreement; this may give a more realistic indication of the performance of similar physical activity questions in an epidemiological setting. As our findings and those of others [5,24,25,28,29] indicate that repeatability decreases with time, it is important to regularly update measures of physical activity in prospective studies [14].

The main strengths of this study include the large sample size and prospective study design. A potential limitation of this work is the use of Ainsworth *et al*.'s [19] compendium to estimate METs and the calculation of estimated excess METs instead of the estimation of 24-hour energy expenditure. While this compendium provides an estimate of the caloric energy expenditure required to perform a variety of different activities at various intensity levels, these values are often based on data from individuals atypical to the general population such as young, active males [1]. Furthermore, as in any epidemiological study there may also be inter-individual variation in caloric energy expenditure resulting from differences in metabolic and mechanical efficiency [1,34], and simple questions cannot capture all aspects of physical activity. Despite illustrating that our two different self-reported measures of physical activity agree well in terms of ability to rank women according to their level of physical activity, we did not have objective measurements of physical activity against which to compare the questionnaire data. We were therefore unable to make direct estimates of the magnitudes of biases in associations between health outcomes and self-reported physical activity data, which are likely to require independent, objective measures of activity levels [31].

## Conclusions

There was generally a good association between basic questions which assess the frequency of any physical activity and of strenuous activity, and the hours of and excess metabolic equivalents estimated from more detailed physical activity questions. Relationships with various physical activities were stronger for walking, cycling, gardening and strenuous physical activities, than they were for housework. As repeatability of reported frequency of physical activity may decrease over time, physical activity data should be regularly updated in prospective studies.

## Competing interests

The authors declare that they have no competing interests.

## Authors' contributions

MEGA participated in the design of the study, performed the statistical analysis, interpreted the data, and drafted the manuscript. BJC helped to draft the manuscript, helped in the statistical analysis and critically revised the manuscript. JG critically revised the manuscript. GKR critically revised the manuscript. VB conceived of the study, participated in its design and coordination and critically revised the manuscript. All authors read and approved the final manuscript.

## Funding

This work was supported by public funds from Cancer Research UK and the UK Medical Research Council. The funders did not influence the conduct of the study or the preparation of this report.

### Steering Committee

Joan Austoker, Emily Banks, Valerie Beral, Judith Church, Ruth English, Jane Green, Julietta Patnick, Richard Peto, Gillian Reeves, Martin Vessey, and Matthew Wallis.

### NHS Breast Screening Centres collaborating in the Million Women Study (in alphabetical order)

Avon, Aylesbury, Barnsley, Basingstoke, Bedfordshire & Hertfordshire, Cambridge & Huntingdon, Chelmsford & Colchester, Chester, Cornwall, Crewe, Cumbria, Doncaster, Dorset, East Berkshire, East Cheshire, East Devon, East of Scotland, East Suffolk, East Sussex, Gateshead, Gloucestershire, Great Yarmouth, Hereford & Worcester, Kent (Canterbury, Rochester, Maidstone), Kings Lynn, Leicestershire, Liverpool, Manchester, Milton Keynes, Newcastle, North Birmingham, North East Scotland, North Lancashire, North Middlesex, North Nottingham, North of Scotland, North Tees, North Yorkshire, Nottingham, Oxford, Portsmouth, Rotherham, Sheffield, Shropshire, Somerset, South Birmingham, South East Scotland, South East Staffordshire, South Derbyshire, South Essex, South Lancashire, South West Scotland, Surrey, Warrington Halton St Helens & Knowsley, Warwickshire Solihull & Coventry, West Berkshire, West Devon, West London, West Suffolk, West Sussex, Wiltshire, Winchester, Wirral and Wycombe.

### Million Women Study Co-ordinating Centre

Simon Abbott, Miranda Armstrong, Krys Baker, Angela Balkwill, Vicky Benson, Valerie Beral, Judith Black, Anna Brown, Diana Bull, Benjamin Cairns, Dexter Canoy, James Chivenga, Mary Kroll, Barbara Crossley, Gabriella Czanner, Dave Ewart, Sarah Ewart, Lee Fletcher, Toral Gathani, Laura Gerrard, Adrian Goodill, Jane Green, Isobel Green, Joy Hooley, Sau Wan Kan, Carol Keene, Oksana Kirichek, Nicky Langston, Maria-Jose Luque, Lynn Pank, Kirstin Pirie, Gillian Reeves, Andrew Roddam, Emma Sherman, Moya Simmonds, Elizabeth Spencer, Helena Strange, Sian Sweetland, Alison Timadjer, Sarah Tipper, Joanna Watson, Stephen Williams, Lucy Wright.

## Pre-publication history

The pre-publication history for this paper can be accessed here:

http://www.biomedcentral.com/1471-2288/11/97/prepub
